# The development and evaluation of an HIV implementation science network in New England: lessons learned

**DOI:** 10.1186/s43058-021-00165-2

**Published:** 2021-06-10

**Authors:** Jacob J. van den Berg, Elaine O’Keefe, Daniel Davidson, David A. Fiellin, Trace Kershaw, Russell C. Barbour, Susan Cu-Uvin, Paul D. Cleary

**Affiliations:** 1 Department of Behavioral and Social Sciences, Center for Alcohol and Addiction Studies, School of Public Health, Brown University, Providence, RI USA; 2 Department of Epidemiology, Harvard T.H. Chan School of Public Health, Boston, MA USA; 3 Center for Interdisciplinary Research on AIDS, Yale School of Public Health, PO Box 208034, New Haven, CT 06520-8034 USA; 4 Department of Medicine, Yale School of Medicine, 333 Cedar Street, New Haven, CT 06520 USA; 5 Department of Social and Behavioral Sciences, Yale School of Public Health, 60 College Street, New Haven, CT 06520-8034 USA; 6 Department of Ob-Gyn and Medicine, Alpert School of Medicine, Brown University, Providence, USA; 7 Division of Infectious Diseases, The Miriam Hospital, Providence, RI USA; 8 Department of Health Policy and Management, Yale School of Public Health, 60 College Street, New Haven, CT 06520-8034 USA

**Keywords:** HIV, Implementation science, Primary prevention, Cities, Community-based participatory research

## Abstract

**Background:**

Describe and evaluate an implementation science network focused on HIV prevention and treatment in New England.

**Methods:**

In 2014, we established a partnership among university researchers and community stakeholders to stimulate and support HIV-related implementation research. We solicited information from Network members through surveys, interviews at Network events, and dialog with participants. In 2017, we conducted a sociocentric network assessment of collaborations on research projects, grants, manuscripts, and consultations.

**Results:**

We identified 988 connections made through the Network that resulted in 185 manuscripts published and 15 grants funded. Our experience indicated that eight factors were instrumental in building and sustaining the Network: (1) acknowledging different perspectives, (2) balancing content and expertise, (3) encouraging consistent engagement, (4) providing seed funding, (5) membership flexibility, (6) maintenance of Network interactions, (7) supporting local HIV prevention and treatment efforts, and (8) maintaining productive relationships with health departments and community-based organizations.

**Conclusions:**

Developing and maintaining a regional network on implementation science for HIV prevention and treatment is feasible and can facilitate new and productive partnerships among researchers and community organizations and members.

Contributions to the literature

- ▪ HIV implementation science focuses on translating evidence-based interventions into policy and practice for improving the quality and effectiveness of HIV prevention and treatment efforts.
- ▪ A regional network centered around HIV implementation science is an effective way to establish and support collaborative partnerships among agencies, stakeholders, and researchers to address the high prevalence of HIV in small urban areas in New England.
- ▪ Lessons learned in forming and maintaining this type of network can help to encourage and inform the development and continuation of regional networks on HIV implementation science in other parts of the USA and world.

## Background

Promoting the implementation of prevention and treatment interventions that have been shown to be effective is urgently needed [1]. Implementation science has been defined as “the study of methods to promote the adoption and integration of evidence-based practices, interventions, and policies into routine health care and public health setting.” [2]. Implementation science plays an important role in identifying barriers and facilitators of effective health programming and policymaking [2].

In the fields of HIV prevention and treatment, there is growing interest in implementation science [3]. It fills gaps between the perspectives of researchers, clinicians, and public health practitioners by evaluating the use of evidence-based interventions in clinical and community settings [4]. HIV implementation science is a multidisciplinary specialty that seeks generalizable knowledge about the behavior of stakeholders, organizations, and communities to close the gap between evidence and routine practice for health in real-world contexts [5].

The persistent HIV epidemic in small urban cities has heightened interest in implementing effective interventions as these areas face unique challenges to implementation, including smaller social networks, increased stigma, fewer specialized resources, and migration in and out of rural and large urban centers [6, 7]. HIV prevention and care interventions that are designed for larger urban centers but then applied to small urban areas, may not translate well.

The Yale Center for Interdisciplinary Research on AIDS (CIRA) and the Providence/Boston Center for AIDS Research (P/B CFAR) collaborated in 2014 to develop a New England HIV Implementation Science Network (hereafter referred to as the Network) to share regional knowledge to improve HIV prevention and treatment efforts in small urban areas defined as cities with a population less than 200,000. The goals of the Network were to (1) foster partnerships among agencies, stakeholders, and researchers and (2) stimulate and support research and evaluation collaborations across New England, with a focus on implementation science in small urban areas with a high prevalence of HIV. In this report, we describe the establishment of the Network, the lessons learned, and its impact.

## Methods

### Network symposia

To form the Network, Yale CIRA and P/B CFAR identified and invited stakeholders, including all regional health departments, AIDS service organizations, HIV providers and clinics, Federally Qualified Health Centers (FQHCs), academic institutions, patient advocacy groups, industry representatives, and non-governmental research organizations to become Network members. To date, the Network has conducted a total of six annual symposia, four supplemental workshops, and multiple leadership planning meetings (Table 1). Attendance at these gatherings generally included members from community-based organizations (CBOs) (29%), research institutions (29%), community health centers (17%), regional health departments (11%), other (8%), advocates (4%), and industry (2%). After each event, CIRA and the P/B CFAR facilitated follow-up meetings to support research collaborations for grant applications on HIV implementation science.

**Table 1:** List of symposia and selected workshops from the New England HIV Implementation Science Network, 2014–2019

#	Themes	Topics	Presentation examples	Dates	Locations	Attendance
1	Symposium 1: “Network Launch”	– Network vision and rationale – Overview of HIV in small urban areas in New England – Identifying priorities for HIV implementation science – Selected HIV research projects from small and large urban areas – Opportunities for practice and research collaborations	“HIV prevention needs among MSM in small urban areas”	6/4/14	Mystic, CT	160
2	“Research Interest Group Meetings”	– Defining key research priorities for four interest groups: hard-to-reach/high-risk populations; mapping; modeling and cost utility analysis; technology and social media – Fundamentals of implementation science for research proposals	“Developing a Common Framework: Key Implementation Science Ingredients for our Network” “Overview of Implementation Science Frameworks with Examples”	2/27/15	Sturbridge, MA	68
3	Symposium 2: “Concepts to Projects”	– Spotlight on regional HIV implementation projects in Network – Moving from clinical trials to community PrEP implementation – Developing Network partnerships to effectively implement PrEP – Creating new regional HIV implementation projects in Network	“Developing a comprehensive model of the HIV care continuum in nine small cities in CT, MA, and RI”	6/3/15	Sturbridge, MA	120
4	Symposium 3: “Successes and Advancing Projects”	– Spotlight on regional HIV implementation projects in Network – Using the Network to develop successful implementation science grant proposals – Concurrent themed discussion sessions on HIV priority groups	“Working with HIV clinics to adopt addiction treatments using implementation facilitation (What IF?)”	6/2/16	Mystic, CT	125
5	Workshop: Using Implementation Science Methods	– Utilizing implementation research frameworks – Developing project ideas with implementation researchers, practitioners, and policymakers – Relevant funding announcements and strategies to respond	“Using Implementation Science Methods in HIV/AIDS Prevention and Treatment Research”	1/17/17	New Haven, CT	48
6	Workshop: Community Research Capacity Building	– Building the capacity of community partners in research and program evaluation methods – Identifying strategies to disseminate and utilize research/evaluation findings – Developing project ideas with implementation researchers, practitioners, and policymakers	“Framing a research question that matters and can be answered”	3/30/17	Sturbridge, MA	24
7	Symposium 4: “Putting Implementation Science into Practice”	– Network analysis of connections/collaborations among members – Updates on HIV continuum of care from New England states – Approaches to community-academic partnership formation – Selecting an IS research framework – Developing pilot project ideas into collaborative proposals	“Operationalizing implementation science in research projects: selecting and using conceptual frameworks” “Research Project Development – Finding the Right Partners, Interventions, and Implementation Science Framework”	5/25/17	Mystic, CT	79
8	Symposium 5: “Community Research Priorities”	– Updates and lessons learned from Network affiliated projects – Exploring development of HIV implementation research proposals based on community priorities	“Turning service needs into implementation projects: examples from the Network”	5/30/18	Worcester, MA	56
9	Symposium 6: “Implementation Science Capacity Building”	– Network overview and network project updates – Workshops on implementation research grantsmanship, program evaluation, and community engagement – Collaborative research proposal development	“Grantsmanship: Using the INSPECT Framework to Strengthen your Implementation Science Grant Proposal” “How to Remain Fidelity-Consistent in Local Adaptations of Evidence-Based Interventions”	5/31/19	Providence, RI	71

*MSM* men who have sex with men, *PrEP* pre-exposure prophylaxis, *CT* Connecticut, *MA* Massachusetts, *RI* Rhode Island

### Network goals

**Fig. 1:**
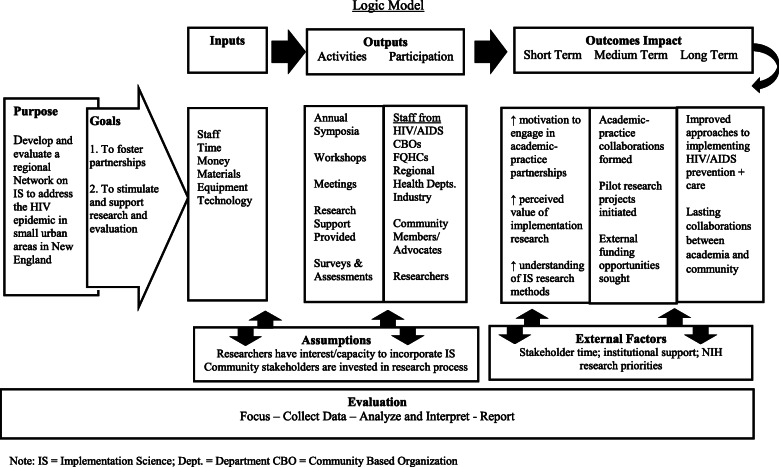
New England HIV Implementation Science Network Development: planning, implementation, and evaluation

Our approach to achieving Network goals included (1) developing an understanding of implementation science research concepts; (2) forming academic-community partnerships around topics of mutual interests; (3) obtaining input from community partners on local needs/priorities; (4) generating research questions that could be addressed through implementation science research; (5) offering support to academic-community partnerships interested in pursuing research funding by linking them to implementation science experts, providing logistical meeting support, and providing seed funding; and (6) encouraging Network members to report their experiences and successes to inform future Network partnerships. Figure 1 presents a logic model of the relationships between the network purpose, goals, inputs, outputs, and impact.

### Capacity building efforts

The Network has provided training on implementation science and supported the development of collaborative projects by creating an online Network Resource Center, establishing a joint pilot project award supported by the two centers, disseminating grant opportunities and facilitating discussions to incentivize collaborations, promoting implementation science capacity through webinars, the aforementioned workshops, facilitating matchmaking of researchers and with community/practice partners, and convening meetings to coordinate and refine research project concepts.

### Assessment

Quantitative and qualitative feedback was routinely solicited from members about the organization, logistics, and quality of Network activities through electronic surveys, verbal feedback at Network events, informal interviews, and periodic dialog with representatives from the research and practice spheres.

At the fourth Network symposium in 2017, we undertook a more systematic evaluation of its impact on building collaborations and projects by conducting a sociocentric network assessment [8]. We asked 84 of the most active members of the Network to anonymously describe their ties/connections with other Network members on (a) research projects, (b) grants submitted and received, (c) manuscripts, and (d) consultations. Individual responses were aggregated within organization and analyzed interactions by organization and organization type (e.g., research, health department, community-based organization) [9, 10]. Social network analysis was used to assess Network characteristics, including density (ratio of the number of observed ties divided by the total number of possible ties) [10]. Seventy-five individuals from 34 organizations completed the social network assessment. Lessons learned were generated from open ended feedback and process notes taken by staff that were discussed by the leadership group until consensus of the main lessons learned were reached.

## Results

In almost all Network meetings, researchers comprised less than half of the attendees. After the initial “kick-off” meeting, participants identified their primary areas of interest and four workgroups were formed around different topics (e.g., hard-to-reach/high-risk populations, technology and social media, modeling and cost utility analysis, and mapping) to develop research ideas. A major goal of the second symposium was to move from “Concepts to Projects.” A third symposium focused on advancing emerging ideas. Subsequent workshops focused on implementation science methods, mathematical modeling, and developing community research capacity. The fourth symposium focused on “Putting Implementation Science into Practice,” while the most recent symposia focused on addressing topics of interest to community participants and providing a mix of capacity building activities to better meet the needs of participants with varying levels of implementation science expertise. The symposia had multiple functions in addition to capacity building and the Network did not provide a structured curriculum on implementation science. Nevertheless, all meetings addressed issues in implementation science to some extent. The issues and topics covered are summarized in Table 1. One of the goals of the Network was to provide resources to support the development and execution of implementation science initiatives. To that end, CIRA developed a website where Network information is compiled (https://cira.yale.edu/network); it includes a wide range of resources related to implementation science.

The sociocentric network assessment documented 988 connections between organizations that resulted in 185 manuscripts published and 15 grants funded. Most connections with research institutions in the Network were with non-academic, implementing organizations. After eliminating multiple connections, there were 579 unique connections from network members at research institutions. Of those, 308 were to implementing organizations, such as health departments, FQHCs, CBOs, and to a lesser extent industry.

There were 285 connections generated by Network activities related to research ideas; 200 connections resulted in grant submissions, and 162 connections represented grant-funded activities. Eighty ties represented co-authorships on a manuscript. Using a standard measure of network density [8], the ratio of observed pairings to all possible pairings (8128) yielded a value of .15, suggesting a very high density.

Network activities led to 16 collaborative research projects as listed in Table 2. Some projects were supported by CIRA and/or P/B CFAR pilot funds, others were submitted as National Institutes of Health (NIH) R01 applications with two of those successfully funded, and several with more immediate practical application were funded by state health departments.

**Table 2:** Collaborative research projects across the New England HIV Implementation Science Network, 2014–2019

#	Project title	Implementation science methods or frameworks used	Academic institutions represented	Community or government agencies represented
1	“Adapting an Evidence-based Intervention for Stigma-related Stress, Mental Health, and HIV-risk for MSM of Color in Small Urban Areas”	Adaptation of existing evidence-based intervention (EBI)	1	1
2	“Developing Comprehensive Model of the HIV Continuum of Care in Nine Small Cities in CT, MA, & RI”	None reported	2	3
3	“Assessing Local HIV Care Continuum Experiences in Small Cities”	None reported	2	0
4	“Implementation of HIV PrEP for MSM With and Without Substance Use in Providence, RI and New Haven, CT”	RE-AIM	2	0
5	“PrEP Implementation and Evaluation Among MSM Who Use Substances”	RE-AIM	2	0
6	“Implementing PrEP in a Family Planning Setting”	Patient education as implementation strategy	1	1
7	“Working with HIV Clinics to Adopt Addiction Treatments Using Implementation Facilitation (WHAT-IF?)”	PARiHS (Promoting Action on Research Implementation in Health Services)	4	4
8	“Syphilis TO PrEP Project (Project STOP): PrEP Navigation and Provider Training”	Provider training and patient navigations as implementation strategies	1	3
9	“ePROMISE: Using Social Media to Improve HIV Prevention and CoC in Young MSM”	ADAPT-ITT to adapt existing EBI; Step-Wedge RCT Design	1	3
10	“Project PS: Multi-State MSM Partner Services Study”	Consolidated Framework and Interactive Systems Framework	3	3
11	“Examining Multilevel System Dynamics Affecting HIV Community Viral Load”	System dynamics modeling	2	8
12	“Evaluating Cost Effectiveness of Naloxone at Syringe Exchange Service”	None reported	1	1
13	“Refining and Validating the Community Research Activity Assessment Tool (CREAT)”	Community priority setting using Delphi Method	11	11
14	“Using a Validated Computer Simulation to Assess HIV prevention Efforts in Connecticut”	None reported	2	2
15	“Promoting HIV-risk Reduction Among People Who Inject Drugs: A Stepped Care Approach Using Contingency Management With PrEP Navigation”	Hybrid Effectiveness-Implementation Type I design; implementation-focused process evaluation grounded in RE-AIM and PARiHS	3	2

The authors reviewed information from routine surveys of Symposium participants, interviews at Network events, and periodic dialog with participants and distilled the following lessons about factors affecting the success of Network activities.

### Lessons learned

#### Lesson 1: Acknowledging different perspectives

In clinical trials, there is an emphasis on random assignment of participants to ensure generalizability and reduce bias. They also tend to have strict eligibility criteria. However, community practitioners are often interested in specific sub-populations and/or factors that impact program implementation in specific settings. Moreover, in implementation science, there often is interest in how inner and outer contextual factors impact the implementation or effectiveness of an initiative, an approach that many traditional researchers are not familiar with. These different perspectives must be acknowledged and effectively managed.

Workgroup meetings benefit from the inclusion of strong facilitators who understand how to focus on researchable questions while respecting the perspectives of individuals and agencies that may have different priorities and timeframes. As one participant reported, “My organization does not have the infrastructure or capacity to execute a study, but the subjects we discussed impact the work we do on a very basic, real level, so it would be great to figure out a way to be involved in a study on the subject” (Health Care/Social Service Provider, 2018 Symposium).

#### Lesson 2: Balancing introductory and in-depth material

Surveys of network participants suggest that it was a challenge to provide an optimal balance of introductory and in-depth material. Researchers appreciated the focus on implementation science methods: “Loved the detailed descriptions of the conceptual frameworks and how they were used!” (Researcher, 2017 Symposium) and “Great examples of D+I [Dissemination and Implementation] frameworks and practical application” (Researcher, 2017 Symposium). However, some participants reported didactic content “too scientific and detailed” (Public Health staff, 2015 workshop), “dry and drawn out” (Advocate, 2015 workshop), or “more academic focused than what I deal with on a day-to-day basis” (Health care/Social service provider, 2015 workshop). Educational programs need to address the different levels of experience with implementation science and how it may be defined and utilized differently. Multi-tiered trainings that start out with broad foundational concepts on implementation science and then get more specific depending on the interest and needs of the target audience members may be one way to balance the level of content and expertise presented.

A roughly equal balance of academic researchers and community participants ensured that both were represented in smaller breakout groups, where the goal was typically to meet others with a common interest and to develop potential research questions. These sessions provided for a “Great exchange of ideas” (Advocate, 2015 Symposium) and forged connections, helping attendees to “network with regional colleagues” (Public Health Staff, 2015 Symposium) and “identify other individuals interested in the same questions that I would like answered” (Physician, 2016 Symposium).

#### Lesson 3: Encouraging consistent engagement

Ongoing and frequent engagement with a large, diverse membership is needed to maintain long-term interest in and connection to the Network. Long-term interest in and connection to the Network in turn facilitate capacity building in implementation science. Long-term engagement can also facilitate the development of implementation science research projects. Inclusion of all New England states has been desirable to identify and address similar issues across the region as well as to leverage resources, but involvement in in-person events by the more geographically remote of the six states (e.g., Maine, New Hampshire, Vermont) has proven difficult. Attempts to conduct workgroup meetings by video conference were very challenging because of inconsistent connections and audio quality. Eventually, that approach was dropped, except for very focused, engaged groups. Those meetings were conducted before the widespread use of videoconferencing during the COVID-19 pandemic so this may be much less of a concern for future activities. Large, inclusive meetings are important to establish connections and promote openness, but to achieve results it is necessary to select more engaged members for more focused discussions.

#### Lesson 4: Providing seed funding

Providing seed funding to launch research collaborations for larger studies is essential to move Network member ideas to action and to create mini “learning collaboratives” to cultivate practice-academic research relationships. Time is a key constraint reported by both academic and community participants, and small grants have supported some of the researcher effort required to pursue projects developed during Network events or within Network collaboratives. For example, a “Syphilis to Pre-Exposure Prophylaxis (PrEP)” health navigation project for men who have sex with men (MSM) with syphilis diagnoses was developed during the 2015 Network Symposium with funding from the Connecticut (CT) Department of Public Health. This project was motivated by the rise in number of syphilis cases in the state reported by the local health department and interest in addressing this public health concern among medical practitioners and researchers. These shared interests were discussed at the Network meeting. The experience and connections made among CIRA researchers, health department staff, and local providers stimulated further PrEP linkage efforts in the state and laid the foundation for a successful application for a Centers for Disease Control and Prevention demonstration project funding to CT Department of Public Health in 2018 to promote PrEP adoption among Black/African American and Hispanic/Latino MSM.

#### Lesson 5: Permitting membership fluidity

Network member assessment, surveillance, recruitment, engagement, and re-engagement are an ongoing process that requires careful attention. Membership is fluid and participation can be erratic; people step in and out of the process depending on level of interest, role in the Network, and what is on the agenda that is of interest to different stakeholders. As new people are hired for practice and HIV research positions, and/or different organizations enter the scene due to HIV grant program/funding volatility and other developments, they need to be identified, informed about the Network, and engaged if interested. The experience of existing members also requires evaluation to assess interests/needs for additional support from Network coordinators. It also is important to assess and develop the implementation science skills of members to support advancement of large, funded implementation science research projects.

#### Lesson 6: Maintenance of network interactions

Network maintenance and facilitation require dedicated, appropriately skilled professionals with strong connections to both the practice and research spheres. Building trusting, reciprocal relationships between HIV practice and research sectors also requires having a Network dedicated staff member, and others from Network lead organizations actively involved in the practice sphere, such as attending HIV statewide and community-based meetings in the region, serving on committees, contributing to initiatives such as statewide “Getting to Zero” or “90-90-90” campaigns [11, 12].

#### Lesson 7: Supporting local HIV prevention and treatment efforts

Evaluation of existing local HIV programs is often of interest to HIV clinicians and finding ways to support these needs can bridge the practice-research gap and earn more buy-in from practice partners. These projects are an opportunity to build relationships and community research capacity and can even generate data for use in more research-oriented applications [13, 14]. From the perspective of CBOs, better program data are useful for quality improvement as well as future grant applications. Multiple approaches to engaging and sustaining participation are important, including moving location of meetings, offering large and smaller structured programs, and experimenting with virtual meeting platforms.

#### Lesson 8: Developing sustained and productive relationships

Developing sustained and productive relationships with state health departments is essential. For example, the 2017 Network symposium included a panel of HIV program directors from the six New England state health departments who presented HIV continuum of care data for their areas and identified research questions of regional interest. This led to a three-state Network study designed to examine issues around the approach to and uptake of partner referral services among MSM living with HIV.

Cultivating and sustaining partnerships with CBOs is equally important as they are the front line of the HIV service delivery system where implementation of most HIV interventions occurs. These providers face challenges in selecting and adapting interventions to optimize prevention and treatment outcomes, challenges that can be addressed through implementation science.

## Discussion

In this report, we describe the development and functioning of a new regional model for integrating community and academic efforts in implementation science to address HIV prevention and treatment. The Network has stimulated and supported new collaborative research addressing issues of HIV prevention and treatment in small urban areas and enhanced the research capacity of affiliated scientists and community partners to conduct implementation science.

Our experience demonstrates that a multidisciplinary and multi-sector research network can foster productive and lasting collaborations between researchers and practitioners with tangible outcomes. To optimize these gains will require sustained and strategic support and facilitation of the process, ongoing information sharing, and regional networking. In the future, we plan to disseminate the lessons learned while expanding the Network and to share successful methods for maintaining collaborative partnerships. We hope to expand engagement to interested parties from other regions of the country through the Department of Health and Human Services’ Ending the HIV Epidemic initiatives. Going forward, dissemination approaches will include publications, conference presentations, and the online Network Resource Center. The Network will support the development and evaluation of initiatives that advance the federal government’s National HIV/AIDS Strategy by supporting the development of interventions tailored to small urban centers and other understudied communities.

Now that the Network is running effectively, we will focus more on developing federally funded implementation science projects that build on the unique relationships and collaborations fostered by the Network. Addressing the unevenness in capacity and readiness to engage in implementation science among Network members will be critical to our ability to foster successful grant applications and projects. Implementation science capacity is now much stronger at Network academic institutions due to several new training and grant activities. Thus, in the next phase, we will be able to connect Network members to a broader range of training programs for academic researchers and community practice partners in implementation science methods and community-engaged research. As these programs are refined, we will offer them broadly using digital learning technology.

To reach a broader audience, we will also expand our online Network Resource Center, providing wide access to materials for those interested in conducting research in small urban centers. The Resource Center (https://cira.yale.edu/network) provides (1) links to training on implementation science; (2) information on current implementation, dissemination, and translation research, using social media/technology; (3) bibliographies; and (4) approaches to address challenges in doing research in small urban areas.

## Conclusions

Implementation science has a potentially important role in advancing the goals of the National HIV/AIDS Strategy and the new federal plan to end AIDS by 2030. There is a long history of collaboration among researchers in multiple disciplines [15] and there are often formal organizational structures for such collaborations [16], but implementation research is embedded in community and care sites. To be successful requires the engagement and collaboration of community partners who work in these settings. There are many types of research networks [17], but a network form that has attracted increasing attention is the research consortia, the purpose of which is to conduct research that meets the needs of users and enables them to act [16]. Our experience with the New England HIV Implementation Science Network suggests that such networks can provide a useful platform to support research examining the implementation of interventions and programs addressing HIV prevention and treatment in small urban areas with a high burden of disease that are often understudied.

## Data Availability

The data used and/or analyzed during the current study are available from the corresponding author on reasonable request.

## Abbreviations

CIRA: Center for Interdisciplinary Research on AIDS
CBO: Community-based organization
CT: Connecticut
D+I: Dissemination and Implementation
FQHC: Federally qualified Health Center
HIV: Human immunodeficiency virus
MSM: Men who have sex with men
Network: New England HIV Implementation Science Network
NIH: National Institutes of Health
P/B CFAR: Providence/Boston Center for AIDS Research
PrEP: Pre-exposure prophylaxis
